# Fully Automatic Camera for Personalized Highlight Generation in Sporting Events

**DOI:** 10.3390/s24030736

**Published:** 2024-01-23

**Authors:** Robbe Decorte, Jelle De Bock, Joachim Taelman, Maarten Slembrouck, Steven Verstockt

**Affiliations:** IDLab, Ghent University—imec, Technologiepark-Zwijnaarde 122, 9052 Ghent, Belgium; jelle.debock@ugent.be (J.D.B.); joachim.taelman@ugent.be (J.T.); maarten.slembrouck@ugent.be (M.S.); steven.verstockt@ugent.be (S.V.)

**Keywords:** personalized clips, highlight generation, sensor tracking, video enrichment, sports data science

## Abstract

Personally curated content in short-form video formats provides added value for participants and spectators but is often disregarded in lower-level events because it is too labor-intensive to create or is not recorded at all. Our smart sensor-driven tripod focuses on supplying a unified sensor and video solution to capture personalized highlights for participants in various sporting events with low computational and hardware costs. The relevant parts of the video for each participant are automatically determined by using the timestamps of his/her received sensor data. This is achieved through a customizable clipping mechanism that processes and optimizes both video and sensor data. The clipping mechanism is driven by sensing nearby signals of Adaptive Network Topology (ANT+) capable devices worn by the athletes that provide both locality information and identification. The device was deployed and tested in an amateur-level cycling race in which it provided clips with a detection rate of 92.9%. The associated sensor data were used to automatically extract peloton passages and report riders’ positions on the course, as well as which participants were grouped together. Insights derived from sensor signals can be processed and published in real time, and an upload optimization scheme is proposed that can provide video clips for each rider a maximum of 5 min after the passage if video upload is enabled.

## 1. Introduction

Organizers of sporting events consistently seek ways to enhance brand loyalty [1]. A first step in achieving this objective involves elevating the engagement level for both spectators and athletes. Additional race coverage and content generation not only attract more attention and spectators to the event but also serve as incentives for participation, thereby positively impacting the revenue stream [2].

While strides are being made in traditional broadcasting to align with new media trends, such as personalized short-form video content for effective communication with younger audiences, events with lower budgets, such as youth races or non-televised sports, still lag in this domain. In these cases, interested parties, like parents, fans, and family, often lack access to real-time performance updates, relying instead on others to convey information, despite the availability of tracking technology [3,4,5,6]. Participants may desire action shots for social media, but the current reliance on roadside photographers or spectators with mobile phones poses limitations. Even in broadcasted races, finding specific participant clips remains a manual and time-consuming task. This manual process not only hampers the creation of timely highlights but also limits the potential for delivering updates on an individual’s position in the race, whether in real time or post-event, which could be valuable for fans and sports federations, providing insights into challenging segments and athlete performance.

The issue of fragmented video content storage further complicates content discovery (multiple sources), as there is no standardized platform for uploading available videos. Automation of this process necessitates provision of additional metadata, such as who appears in the video, which is a time-consuming task. In addition to video-driven platforms, textual updates, including the number of groups in the race, intermediary lap times, and lists of participants who are out of the race, are only available post-race through manual processing of timing system data. Metrics that are harder to come by may possibly be reported through subjective and sometimes incomplete memories about the event situation. Automated systems could streamline the reporting of such metrics based on objective measurements during the race.

While efforts by racing organizations have increased data availability for high-level races, the solutions are often impractical for lower-budget races. An example at the highest level is the collaboration between Nippon Telegraph and Telephone Corporation (NTT) and Amaury Sport Organization (A.S.O.) at the Tour de France, which involves sensors generating 2.5 million data points per stage to enrich broadcasting streams [5,7]. However, leveraging this data for personalized clips still requires substantial effort from race organizers.

**Figure 1:**
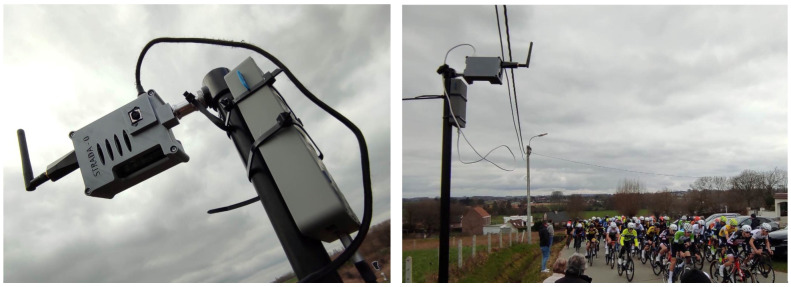
Hardware setup of a STRADA device during junior cycling race field test in Sint-Maria-Lierde.

**Figure 2:**
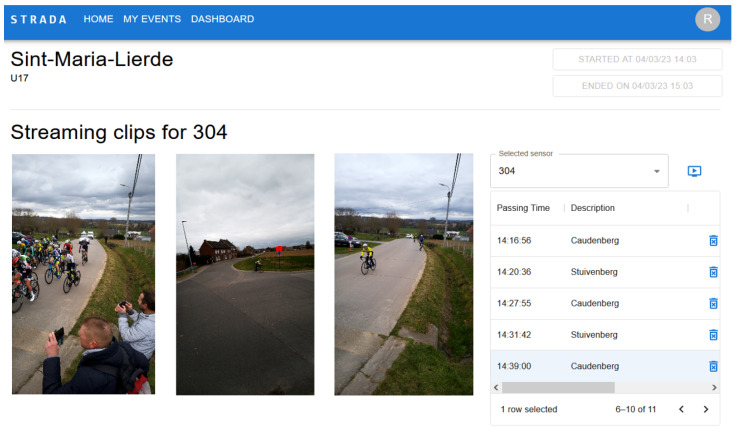
Sample view of a user consulting the generated highlights of an event. To the right of the video player, a selection menu can be used to select which clip to watch, which also shows when the clips were registered and by which device. In this case, the clips were coupled to the sensor with ID 304 worn by a rider with a yellow jersey.

In this work, we present an automated workflow that combines video footage and sensor data to generate low-cost personalized clips. Our setup is capable of reporting changes in the race progression as well as general information about the participants (intermediary times, relative position, etc.). The video and sensor data are captured through a Sensor-driven Tripod for Recording Athlete Data (STRADA). All derivatives are calculated from the data provided by the STRADA devices. The purpose-built device is shown in Figure 1. The goal of this research is to provide race participants with a platform (see Figure 2) that automates the processing and finding of video clips, as well as all the necessary hardware to facilitate the video footage and identification metadata. In the particular case shown in Figure 2, the platform shows all available clips for the athlete that used the sensor with ID 304 and participated in the 13th Grote Prijs Peter Van Petegem on 4 March 2023.

The remainder of this paper is organized as follows: Section 2 discusses relevant related work focused around automated highlight generation in various sporting events and sensor-based tracking. In Section 3, the required hardware setup is presented with relevant information about the configuration process and the workings of the internal software. The section describes the flow from the STRADA device to the central server where individualized video clips are generated on a custom streaming platform. Section 4 discusses the results from a field test during a junior cycling race. The core contributions and general findings regarding the clipping solution are summarized in Section 5, and the next steps to extend and improve the applicability of our solution are described.

## 2. Related Work

### 2.1. Individualized Sensor-Based Tracking

Our device needs to pick up sensor data when sensors are in close proximity. Therefore, we first explore existing methods to achieve this goal. One possible approach is to further utilize existing timing systems, as they require athletes to be within a certain range of a receiver to be detected. These systems typically employ wireless communication protocols and require athletes to wear active or passive tags. In large-scale sporting events, radio-frequency identification (RFID)-based detection is prevalent [8,9]. A study by Ehlerova et al. [10] demonstrated the effective use of ultra-high-frequency RFID tags in various sports. The tags are detected through a receiver positioned in an overhead banner along the race course. The additional hardware required for the athletes is minimal and provided by the organizers. Some opt to incorporate the RFID tags in stickers attached to their bib numbers, while others opt for reusable and clippable hardware pods. Although the impact on the athlete is limited, organizers do have to provide the registration checkpoints. These cannot always be easily incorporated into the course and adding extra scaffolding to accommodate them adds more costs to an already costly system (https://www.mylaps.com/active-sports/running/running-pricing/, accessed on 21 October 2023). This implies that a participant would get a new ID associated with their RFID tag for each event that they attend, which would be harder to maintain for a central collection platform that is not linked to one specific event/organization. Also, this setup only provides timing information because the transmitted signals only include a means of identification. The device presented in this paper can reuse performance-oriented sensors that are already being worn (low burden of entry). In addition to a means of identification, the sensors can also report extra sports-related parameters, such as heart rate, including them in the textual updates during the race. Another technique described by Fasel et al. [11] introduces a magnetism-based timing system for downhill skiing. By placing magnets in the flag poles and equipping skiers with magnetometers, it is possible to detect disturbances in the magnetic field as they pass by the poles. The study utilizes purpose-built magnets to enhance timing accuracy by reducing the detection range. However, stronger magnets can be employed in applications where accuracy beyond 0.25 s is not a limitation. One significant shortcoming of this setup concerning our research context arises when attempting to enable real-time detection. Detected peaks should be linked to an individual athlete, but in sports where multiple athletes can pass the detection point in a group, this is not possible. One partial solution involves deploying base stations along the course that connect with the athlete’s magnetometer. However, the duration of time that the device is in range may be too limited to establish a three-way handshake connection for many sports, especially skiing.

**Figure 3:**
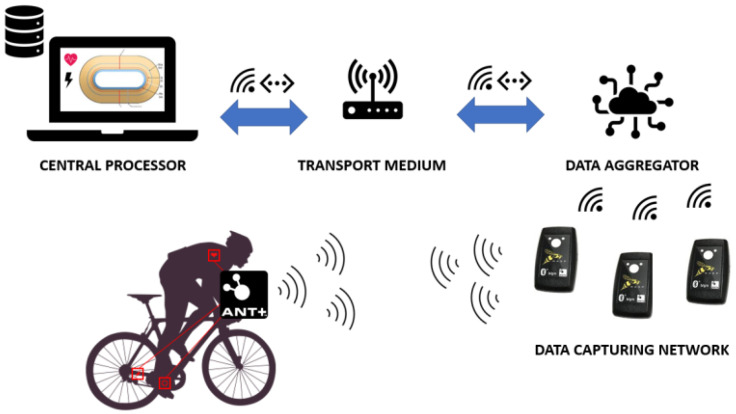
Schematic overview of sensor data flow using WASP-N devices [17].

Advancements in wearable sensor technology have led to the emergence of low-power sensor networks, commonly referred to as wireless body sensor networks [12,13]. These networks involve attaching sensors to the body or sports equipment, with a central component responsible for processing and storing various data streams for an individual athlete. This end-to-end connection between a sensor and a central component is used in different commercial products. For example, running pods and heart-rate monitors communicate with sports watches during running events and power, speed, and cadence sensors interact with the head unit on bicycles. The choice of communication technology may vary depending on the specific context. Two widely adopted wireless technologies in the field of sports and fitness monitoring devices are Bluetooth low energy (BLE) and the low-power standard of Adaptive Network Topology (ANT+) [14]. Both operate within the 2.4 GHz ISM frequency band, offering comparable data throughput and range. Identification is provided with each message by using a unique device identifier. However, there are key differences between these protocols. When connecting an ANT+ device with a tracker, the device remains visible to other scanners in the vicinity that support ANT+. This is because an ANT+-capable sensor broadcasts its values. On the other hand, BLE establishes a one-to-one connection. The number of simultaneous connections varies but can be set by the physical layer (BLE controller). If this limit is reached, the device will not be able to transmit data to other devices. Note that BLE can replicate broadcasting behavior by changing the data included in the advertisement packet [15]. This does require a change in firmware and will not work out-of-the-box. Consequently, with ANT+, the transmitted values can be received by third-party devices without interfering with the intended functionality for the athlete. Originally designed for sport equipment, ANT+ is now compatible with a wide range of sports-related sensor types through standardized device profiles [16]. The practical feasibility of using ANT+ for real-time data capturing has been demonstrated by De Bock et al. [17]. They created a data collection network in an indoor cycling track that gives real-time feedback to coaches. In their setup, sensor values are transmitted and collected using specialized devices called WASP-N (https://support.npe.fit/hc/en-us/articles/360033283551-WASP-N-Product-Brief, accessed on 5 September 2023). These devices serve as translation units, bridging ANT+ messages to a local WiFi or Ethernet network. Figure 3 provides an overview of how these components are integrated to capture and centralize athletes’ data. Multiple WASP-N receptors are strategically positioned along the cycling track. Each receptor multicasts the received ANT+ datagrams within a local network. The multicast packets are then received and processed by a central computer, which is connected to the same network and utilizes the application programming interface (API) provided by the WASP-N manufacturers. These devices work well in controllable environments but always require a nearby network, which introduces an extra hardware cost. It is also not as easily incorporated into a single packaged device. They are designed for a central computer that collects data from multiple WASP-N devices. Since, in our setup, the collection and preprocessing are carried out by the same device and devices may be spread out very far away from each other, a lot of functionality would be unused (especially the bridging capabilities). Another drawback is that the computing unit that processes the signals has to run the Windows operating system because alternatives cannot use the provided API.

### 2.2. Video-Based Identification

Tracing participants in sports footage can be accomplished through visual identification methods. Most sports require an identification number on participants’ jerseys. However, automatically recognizing these numbers poses a challenging computer vision task due to pose variations in different sports and diverse recording setups (such as different camera angles, zoom levels, etc.). Text/digit extraction has many practical applications in addition to sports-related contexts and reading text in uncontrolled environments is still actively being researched [18,19,20]. Liu and Bhanu demonstrated the use of region proposal networks to identify areas suitable for number recognition [21]. These regions are classified as either background, player, or digit. Geometrically related player and digit regions, identified through overlapping segmentations and calculated using a proposal association score, are combined into a single bounding box and fed into a digit recognition network. Alternatively, another approach to identify areas likely to contain a number involves searching for body parts where numbers would be placed [22,23,24]. The resulting areas are then further processed by the recognition network. One implementation of this method known as human parsing utilizes the backbone of a ResNet classification network, incorporating Context Embeddings with Edge Perceiving (CE2P) [25] to obtain a segmentation mask of different body parts. Sportograf provide a practical example of implementing this idea into a service. For mass sporting events, they offer number recognition services for bib numbers worn during the event. Sportograf assign photographers along the race course, and participants can subsequently search for their bib number or upload a selfie (see https://helpdesk.sportograf.com/en/support/solutions/articles/77000538006-search-by-selfie-face-recognition (accessed on 23 August 2023) for facial embedding search). Once a number is associated with an individual, it enables automated searching of associated pictures without human intervention [26].

However, the applicability of these solutions depends on how the sports footage is captured. For static cameras with a fixed zoom during the recording, it may not be possible to view each bib number adequately if the resolution is too low. For sports where participants find themselves in larger groups close to each other, occlusion poses an extra challenge. Therefore, to cover most of the participants, multiple cameras would be required or a human operator who tries to find the optimal viewer experience, but for low-budget events, this is often not feasible.

### 2.3. Highlight Generation in Sporting Events

Historically, there has been much research conducted into video summarization of sports broadcasts. With the growing amount of available video information, organizers can be quickly overwhelmed. Therefore, automatic detection of semantically important events in video and further summarization of video to help the indexing, browsing, and consuming of the video have become increasingly important [27]. Highlight generation can be seen as a subtask in summarization and can be further divided into triggers based on events and excitement [28]. Machine learning-driven video summarization in sports has emerged as a transformative tool, streamlining the process of distilling lengthy sports footage into concise and engaging summaries. These systems can automatically identify key events, highlights, and pivotal moments within a game/race. For instance, a deep neural network-based approach was used by Tejero-de-Pablos et al. [29] to extract two types of action-related features and classify video segments into interesting or uninteresting parts. De Bock et al. [30] suggested a rider/team jersey recognition tool for cyclocross analysis. It is combined with skeleton-based pose detection, based on Alphapose and extended with a spatiotemporally aware pose tracker, to analyze course traversal (time spent running, chosen path in a sand bank, etc.) and can automatically flag interesting segments based on those quantifiable parameters for a specific rider. The presented results of their approach were obtained using a multi-GPU system for model inference. This is not feasible for edge devices as it would significantly increase their cost and size. Specialized hardware, like the Intel Neural Compute Stick or Google Coral, can mitigate the computational challenges of edge devices, offering efficient and cost-effective alternatives to GPU-based setups for pose-estimation models. These accelerators leverage dedicated neural processing units, reducing both the size and power consumption of edge devices. Although recent findings suggest that lightweight pose-estimation models can also perform this task with limited computational power [31,32], it would still lack an identification mechanism. The mentioned techniques are still relevant, but in our system, we use sensor-based tracking, while the video analysis techniques are kept as postprocessing steps on the central server; for example, smart cropping and generating pan–tilt–zoom (PTZ)-like recordings from static video to obtain a more dynamic clip [33]. In most cases, these techniques do not provide personalized analyses. They can be extended with instance recognition, but even then different instances are not easily linked unless an identification number is visible in the footage.

Based on the literature presented in this section, we can unify different parts to create a personalized clipping algorithm. Sensor-based presence detection is preferred over video-based techniques. Running different video detectors in real time requires more expensive hardware, thus decreasing the scalability of the system. As well as that, the identification numbers of participants should always be visible. Otherwise, the system will not be able to automatically link the clips to the right person. To also accommodate fast-paced sports, the broadcasted messages used in ANT+ are more suited than the BLE protocol. ANT+ also takes preference over RFID since the more accurate timings that RFID provides do not outweigh the extra costs of the registration banner/mat and of providing everybody with a tag vs. reusing already available sensors and capturing the signals with a cost-effective antenna.

## 3. Materials and Methods

**Figure 4:**
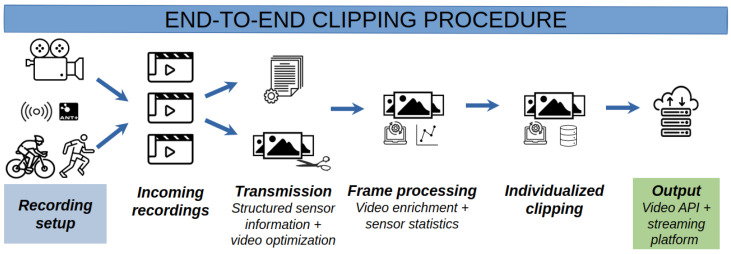
Different steps in the video processing pipeline to produce individualized clips/highlights.

Athletes, recreational or professional, use a mixed collection of sensors on their equipment and on the body. These are primarily used to measure performance during a race or training session, but the broadcasted signals from these sensors can be used to sense when someone is near the STRADA device as well (see Section 2.1). The proposed methodology, shown in Figure 4, consists of several building blocks that lead to the availability of personalized video clips for athletes wearing one or more ANT+-capable sensors. The first step is to deploy the device in an interesting position along the track. The positions can be decided through the personal experience of the organizer or by analyzing the GPS coordinates of the course. Incoming recordings, which we will call video blocks because they have a fixed duration, are prepared for transmission to the central server. This preparation consists of optimizing the amount of video data that needs to be uploaded based on the received sensor values. The sensor values, which include the ID, type, and value, are also stored in a structured format and used as input for the individualized clipping algorithm together with the uploaded video data. The final results (output of the clip algorithm + metadata) are then published on a central streaming platform such that they can be viewed. The remainder of this section elaborates further upon the device used to capture the relevant data, as well as each of the steps that are required in the personalized clip generation.

Sensors that communicate through ANT+ were selected to register when somebody is nearby the device, mainly because of their widespread presence in existing sports equipment, as well as the non-invasive communication mechanism. While BLE may be sufficient for devices that stay close to each other, it does cause some problems if it is used to deduce locality through proximity. Athletes will constantly move in and out the detection zone of the scanner, initializing/terminating the connection while doing so. BLE requires handshaking, which leads to loss of information when a sensor is in range for a limited amount of time. Therefore, ANT+ provides a good alternative with its broadcasting scheme. As the proposed system reuses sensors that are often already worn by athletes, it does not introduce an extra burden for the participants to obtain results. This partially shifts the responsibility from the organizers to the participants, as each participant provides their own means of detection. This also allows permanent setups, like placing a camera on a famous hill where cyclists can log in and download their clips without registering with an organizer who distributes the tags. Athletes are more likely to be more familiar with ANT+-capable sensors, so they can select an appropriate sensor themselves. RFID sports solutions also exist, but they are not as readily available in finished products that can be used by the consumer with limited configuration. The ANT+ directory website provides a list of supported sensors. Some of the main categories are heart-rate monitors, smart watches, bike computers, and activity monitors.

### 3.1. Recording Device Hardware Setup

The STRADA device functions as a modular system centered around a Raspberry Pi model 4B (RPI), which serves as its primary computing unit. Signal reception from sensors is facilitated by connecting an ANT USB-M antenna. Signal processing is performed using a Python implementation of the ANT+ protocol, along with the accompanying USB drivers, both of which are available in a publicly accessible repository fork (https://github.com/s-team-ghent/idlab-ant, accessed on 15 November 2023). The USB connectivity offers a notable advantage in terms of replaceability, contrasting with alternatives that require soldering to a microcontroller. One major disadvantage of using USB-enabled ANT+ antennas is that they do not report the received signal strength indicator (RSSI) values of the sensor signals. This is unlike some of the nRF components (nRF5340 or the nRF52 series), which need to be soldered to be used. Although RSSI values would possibly improve the versatility of the system’s applicability across more situations, it is not a hard requirement for this set-up to work. Video footage is collected through the standard Raspberry Pi Camera Module 3. Precise control over image processing and better access to the internals are achieved by combining functionality from the libcamera and Picamera2 Python libraries. To ensure automated video synchronization between devices, an accurate system clock is needed. When a device is connected to a Network Time Protocol (NTP) server, either in the local network or through the Internet, this is achieved automatically and maintained by the operating system. However, when the compute unit has no means of communication to one of these servers, it loses its accurate representation of time when powered down or through accumulated clock drift. Since the STRADA devices will have to operate in environments without connectivity, they need a hardware clock as fallback. This can be achieved by using a coin battery-powered DS3231 RTC module on the I2C pins on the Raspberry Pi. From the datasheet [34], we know that the module has an estimated clock drift of ±2 parts per million (ppm; every million seconds, the clock will have drifted 2 s); thus, periodically connecting the device to the Internet will reset the built-up drift. The different components are encased in a custom 3D-printed housing and mounted on top of a tripod.

Video is recorded in blocks of 5 min. This fixed duration is a trade-off between the time it takes to transfer/process each block and the delay in reaching the streaming platform/spectator. The camera records video blocks in a continuous loop. This is required for multiple reasons. Firstly, athletes moving towards the device may be in-frame before their sensor signals are picked up by the antenna. This would occur, for example, in instances where the line of sight between the sensor and the antenna is heavily obstructed, as the duration of the detection window size is inversely proportional to the amount of obstruction. Secondly, in order to support duration extension at the start or end of the clip, video from before and/or after the detection interval is needed. For each newly started video block, the starting time is registered through the integrated hardware clock. These timestamps are used in later steps to synchronize video streams from different cameras and to accurately cut specific parts of video using the timestamp values of the incoming sensor signals.

### 3.2. Clipping Algorithm

Once the video blocks and sensor data are available on the server, the data can be fused together as input for the individualized clipping algorithm. The first part of this algorithm only utilizes the sensor data to find the different clip boundaries for a given sensor ID. This is parameterized by five parameters:

- *t_grouping* controls the grouping factor during the aggregation phase of the data. All values that lie within *t_grouping* seconds form a single instance. This will define the initial clip boundaries using a hard limit on the received timestamp values of the sensor data;
- *t_before* then increases or decreases the duration at the start of the previously found instances. If the updated start time exceeds the coverage limits of the videos, it will be replaced with the closest possible value. Note that *t_before* is dynamically altered based on the sensor type to combat the difference in transmit power;
- *t_after* is the counterpart of *t_before* and is used to change the end boundary of the clip. The same explanation from *t_before* is applicable to this parameter;
- *t_max_duration* and *t_min_duration* are used to filter out clips that are too long (i.e., when someone with a sensor is standing stationary close to the setup) or too short to be considered as usable clips (typically at least 2–3 s). This filtering operation is performed before the buffering operation.

**Figure 5:**
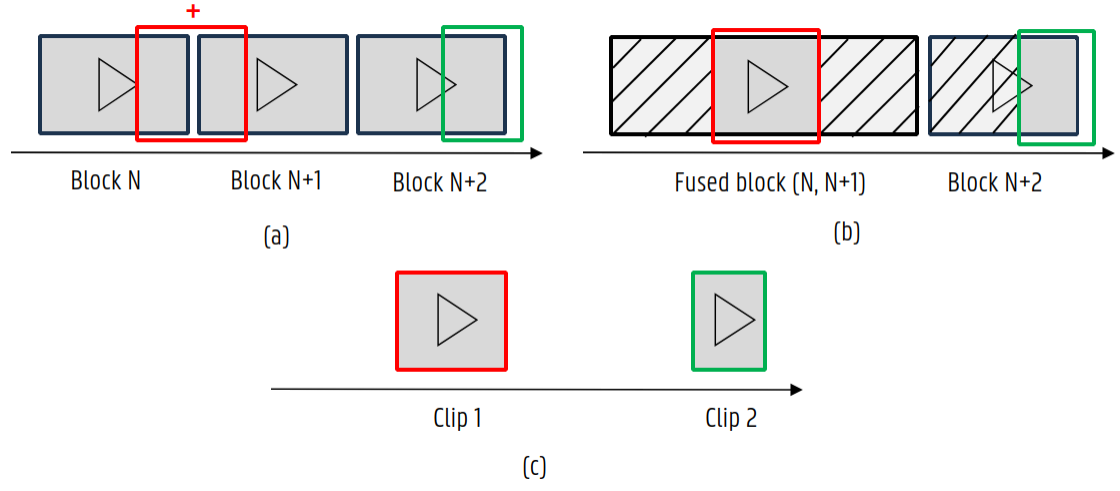
Visual representation of the clipping algorithm. (**a**) Timeline of the transferred blocks on the server. Red and green boxes represent the calculated clip boundaries. As the red clip lies on a video block boundary, an intermediary step is needed. (**b**) If necessary, required video blocks can be combined and unwanted video footage removed according to the clip boundaries. (**c**) Result of the clipping algorithm. As the end boundary of the green block lies outside of the recording window (this may occur if the extension is larger than the max buffer configured on the device), it is clipped to the last available time recorded.

Using the generated metadata, the algorithm can start to select which video blocks are needed to encode the requested video clip. Figure 5 shows a visual representation of the different steps of the clipping algorithm. In case multiple files are needed, slices of each file are created, taking the required buffer period on top of the initial boundaries into account. Previous steps are performed without re-encoding any intermediary steps. Avoiding re-encoding is a considerable speed gain for the algorithm, and re-encoding is at the moment only required when adding virtual overlays to the results or for further compression of the clip.

### 3.3. Video Block Optimization

To facilitate buffering operations preceding or following the reception of sensor signals, the STRADA device operates in a continuous recording mode. The recorded video blocks are subsequently transmitted to the central server for further processing into individualized clips. However, it is important to note that only video data corresponding to the time of the reception of sensor data (inclusive of a buffer interval) are required for clip generation. In online mode, the device transmits sensor data upon arrival and video data once the block is completed. To optimize the upload process, the video block optimizer intelligently minimizes data transfer without losing relevant information. This approach not only reduces upload time but also mitigates long-term evolution (LTE) costs when deployed in the field. Alternatively, the device can operate in offline mode, which is suitable for situations where real-time updates are not a requirement. A hybrid approach is also feasible, wherein textual updates are made possible by uploading sensor data. The processing of video data occurs post-event when a fast and reliable Internet connection is available. This flexible operational mode allows users to adapt the device’s functionality based on their specific needs and connectivity constraints.

The video block optimization procedure leverages some assumptions about the kind of events we wish to record to optimize the length of each video block. Mainly, this concerns whether the amount of sensors in the detection range of the device is constant or whether it spikes and then stays zero for a period of time. Take, for example, a local (lower-level) cycling race. Riders are very often close together in a peloton on a course mostly consisting of multiple and repeating laps. Barring breakaways, riders who have dropped from the main peloton and fallen too far behind are taken out of the race to prevent a fragmented field of participants. This allows the race organizers to secure the bunch while other traffic can flow through (momentarily) unused parts of the circuit. This implies that the passages of each rider will be relatively close to each other and, until the next lap, there will be mostly dead air that is of no use for the clipping algorithm. The device would receive sensor data while the riders pass through the detection zone and then remain idle until the next lap. So, in cases where no sensor signals are received during the duration of one video block, it will not be uploaded. If only a quarter of the block is needed, the other parts will be discarded. This is also the case in other sports. During a training session involving snowboard jumps on a dry slope, we analyzed the amount of movement in the raw video and concluded that only 11% of the recorded video contained footage of the jumps. The amount of movement in the video was calculated using the MOG2 background subtraction algorithm and by counting the number of white pixels. The same procedure was also applied to an endurance exercise in track cycling. In that case, only 16% of the video contained the rider in view of the camera.

The optimization algorithm considers all sensor signals as equals: only their presence is important and not where they originated from. It is initialized with a maximal buffer size (in seconds) for before and after a sensor signal is received. This imposes a new constraint on the clipping algorithm where the selected buffers of a device configuration must lie within the chosen interval of the optimizer. The value of *t_before* lies within $\left[-(\mathrm{clip}\_\mathrm{duration}+\max\_\mathrm{buffer}\_\mathrm{start}),\;\max\_\mathrm{buffer}\_\mathrm{end}\right]$. The same constraint applies to *t_after* when the max start and end sizes are swapped. As before, the device records a block of a set duration, but before uploading, it transforms the fixed-duration block into variable-length blocks by grouping the sensor information received during the current iteration. The algorithm contains a list of timestamp pairs. A new pair (head and tail) is created if a timestamp value exceeds the maximal buffer size or if the list is empty. If a new timestamp lies within the buffer, the tail value is set to the sum of the timestamp and the buffer size. This results in a list with the start and end timestamps for each segment, which can then be used to cut specific parts of the video block.

The available video blocks will no longer be continuous, or at least this cannot be guaranteed. In most cases where there is no relevant sensor data captured during a given block, there will be large gaps in the processed videos that are transferred. The transformed segments will always deliver an equal or net decrease in file size. The performance gain is of course heavily influenced by the use case. More insights regarding the quantification of this procedure are discussed in Section 4. From a user perspective, both methods are functionally equal. The optimization algorithm guarantees that the extra duration of the chosen maximal buffer window is available for every possible sensor value. Note that it may even be longer in a situation where different sensors are picked up with a delay smaller than the buffering window; since the windows overlap, they will all be fused into a single segment. However, this property of longer buffers is not guaranteed and should not be used in later calculations.

### 3.4. Streaming Platform

The users can consult the online streaming platform for all functionalities discussed above. Organizers and athletes both require minimal manual input to configure a new event or obtain highlights. A new event needs a name and a list of STRADA devices. Users only have to link the ANT+ IDs of their sensors used during the event to their account. Discovery of which events someone participated in is fully automated. During the event, when sensor data and recordings are processed by the central server, a user will see a new discovered event as soon as values from his/her registered sensors are received. Organizers use this platform mostly to control the various event parameters as well. They can set the official start and end times of the event, as well as the input parameters for both the recording configuration and the clipping algorithm for each device. Only timestamps of sensor values in that interval are considered for the clip generation. Server-side parameters (see Section 3.2) for each device are also managed through the platform and can be altered after the event has ended, allowing specific clips to be regenerated.

## 4. Results

In the previous section, the building blocks for the individualized clipping mechanism were discussed. In this section, we further elaborate on the implementation of the introduced building blocks and some preliminary results are showcased. The anonymized dataset can be found in an online repository for replication of the experiments (https://github.com/robbedec/datasets/tree/master/STRADA/lierde, accessed on 15 November 2023).

**Figure 6:**
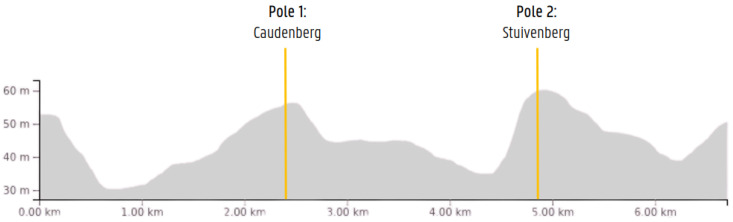
Locations of the STRADA devices projected on the race profile of a single lap.

The setup was deployed for an in-field test during a junior cycling race in Sint-Maria-Lierde (13th Grote Prijs Peter Van Petegem), Belgium. The race consisted of 10 laps of 6.5 km. The positions of the devices, shown in Figure 6, were on the two prominent hills on the course. During the race, which lasted for 1 h and 45 min and included 112 participants, 28,383 sensor values were captured from 378 unique sensors. The distribution of sensor types was as follows: 194 heart rate, 127 power, 59 cadence, and 3 unknown (device profiles not supported by the STRADA device). Some of them were capable of reporting multiple metrics. Before the race, participants could opt into the study by providing the sensor IDs of their sensors, as well as their personal transponder codes used by the timing system of the organizers at the finish line. Out of the 21 registrations, 16 provided a correctly formatted ANT+ ID. To improve this in the future, we plan to design a booth that participants can visit. This will contain a screen that displays the ANT+ IDs of the sensors in the vicinity (preferably limited to those inside the booth).

**Figure 7:**
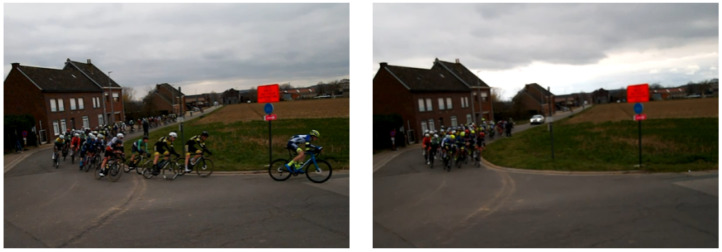
Comparison of peloton density in the Caudenberg section between a fast passage of an elongated peloton (**left**) with riders drafting with two to three people next to each other and a more relaxed passage with riders using a greater portion of the road and thus with a peloton that was not elongated (**right**).

The ground-truth information was obtained by combining the pass-through times after each lap at the finish line for those who signed up for the pilot project and information regarding which collection of sensors belonged to which rider. The time intervals for when the peloton passed by each camera were extracted manually from the pre-optimized video data and are summarized in Table 1. As there was no official classification standard for the peloton during the race (the cut-off for being a backmarker), we utilized the classification metric imposed by the Union Cycliste Internationale (UCI). The UCI provide regulations on how the time gaps are calculated for stages expected to end in a bunch sprint, or in other words, which riders are given the same time because they are considered a homogeneous group [35]. Following this official document, we stopped the peloton timer when the final rider crossed the center line after whom no other rider crossed the line within three seconds. One immediate observation about the peloton passing times was that Caudenberg passages were mostly longer. This was in line with the expectations when considering the course profile and was also verified through the videos. The climb in the Caudenberg section is objectively easier compared to the Stuivenberg section (immediate steepness), and thus quicker speeds naturally transformed into a longer peloton, with most people drafting in two to three lines (after lap five, riders also used a greater portion of the road; see Figure 7), while in the Stuivenberg section, riders were almost fully spread over the whole width of the road.

As mentioned in Section 3.3, the Did Not Finish (DNF) state is a very common occurrence, as everybody too far behind the peloton is taken out of the race. In this race, only 55 participants completed 10 laps, with most DNFs noted in laps three to six, where 21, 9, 10, and 7 occurrences, respectively, were registered. With the individualized lap times available, an overview can be created of how many laps each rider completed. Table 2 and Table 3 contain the durations of the generated clips of the camera in the Stuivenberg (respectively, Caudenberg) section for each lap. The absence of a value in these tables was either due to a failure to detect the sensor values when the lap number was smaller than or equal to the abandonment value or because the participant was taken out of the race. Note that due to a technical issue the Caudenberg device started recording after the first passage. Its table of generated clips therefore contains one column and also one row less because a participant was taken out of the race after the first lap (ID 45538). From these tables, the detection rates can be calculated and correspond to 92.56% for the Stuivenberg device and 93.33% for the one placed in the Caudenberg section.

**Figure 8:**
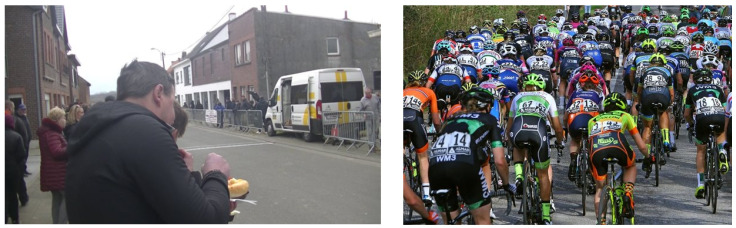
Examples of two common problems when deploying the STRADA device in the wild. Spectators will be oblivious to the effects of standing in front of the camera. A second problem occurs when athletes pass by the device in very large groups, causing sensory overload and blocking each others’ signals with their bodies.

The ability to receive transmitted sensor values is mostly dependent on the distance between the sender and receiver. Empirical testing shows that, with a direct line of sight, the ANT+ antenna can pick up signals from a Garmin chest monitor from approximately 75 m away; without the range extender of hLine antenna, this is reduced to 3–4 m. If we go back to the cycling example, this implies that clips will generally be longer when riders are riding solo or in smaller groups since there is much less occlusion compared to someone in the middle of a peloton. Other factors, such as sensor position and transmission power, also have an impact on clip duration. These problems can be alleviated by positioning the receiver antenna and possibly decoupling it from the camera module to place it at a higher vantage point. This can improve the chance of a less occluded line of sight. With regard to the other problems shown in Figure 8, device position is also vital to obtain quality clips, as people will always find a way to stand in front of the device if they are able to. With larger groups passing the device very close to each other, there will always be more occlusion due to the different bodies blocking the signal. In the case of cycling, the position will also have an impact; for example, power sensors are placed closer to the ground (e.g., in the pedals) compared to heart-rate monitors. To partially overcome this problem, clip extension methods were implemented. These alter clip duration based on sensor type (accommodating differing transmission powers and positions) but will only help if at least one sensor value is picked up during a passage.

**Figure 9:**
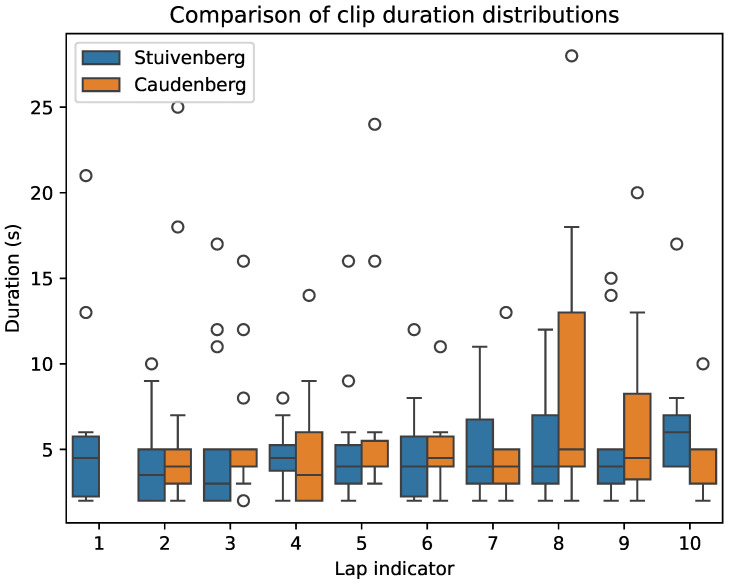
Comparison of the clip durations between both devices for each lap.

In some laps, there was significant variation in clip duration. This is also verifiable through the graph in Figure 9. Intuitively, this indicates that the rider is solo or in a small group such that the signal blocking is limited and/or is moving very slowly. The outliers indicated in the box plot were extracted and are summarized in Table 4 and Table 5. These show, for each outlier, the time difference (in seconds) between the start of the clip and the middle of the peloton passage interval for that lap from Table 1. They also indicate whether the participant was taken out of the race after completing the lap in which the outlier was generated. Following the peloton classification rule of 3 s, it can be concluded that all clips were generated for people who were not part of the peloton. In extreme cases where a rider is multiple minutes behind, the camera information can be used to inform the race organizers how many participants are expected to be taken out of the race at the end of the lap. Reporting this information in real time is also useful to inform spectators about the current race situation.

**Figure 10:**
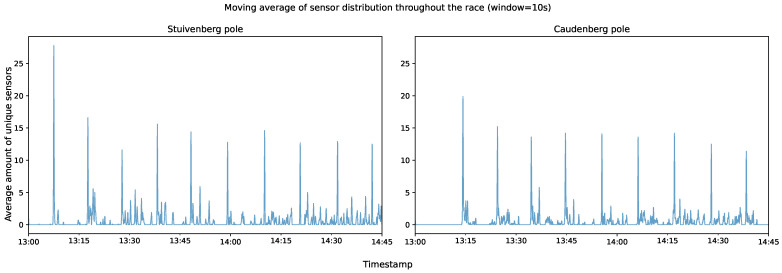
Graphs showing the numbers of unique devices over time during the race. Values were averaged using a moving average of 10 s for visualization purposes. Peaks correspond to the passing of a large group (peloton) and correspond to the number of laps that each device recorded. For a zoomed in portion of a lap, see Figure 11.

**Figure 11:**
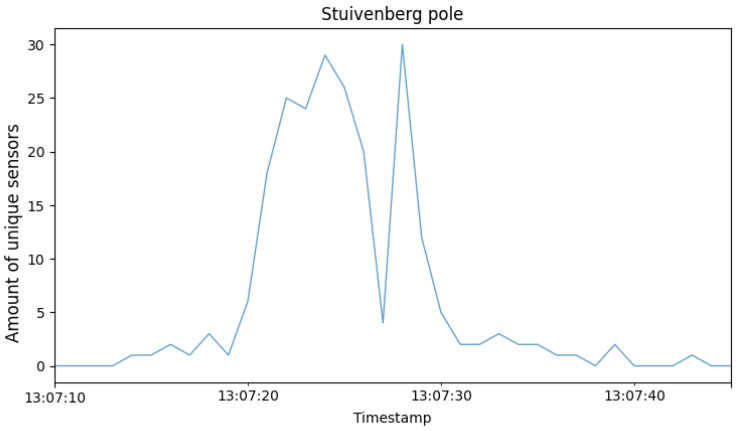
Enlarged portion of the first lap for the Stuivenberg device shown in Figure 10 without averaging. During the passage, riders constantly moved in and out of the detection range.

Although the previous insights utilized the ground-truth times of when the peloton passed the camera, it is also feasible to derive these timings automatically for each lap through the collected sensor data. With these data available, it is possible to answer the research question about how to keep spectators, parents, and others in the loop about position changes during the race. The data are used to create improved textual updates since the system can provide the relative position of the user of a particular sensor compared to the peloton. For this, the full dataset of the sensors is used since it does not require knowledge about sensor–transponder pairs. However, some filtering is advised. For example, some sensors registered 180% to 196% more values (compared to the average) for a particular camera. It is not possible that the owners of these sensors participated in the race since they would have been in frame for a total time of somewhere between 16 and 33 min. A more logical classification for these sensors would be that they belonged to a spectator, perhaps somebody on their Saturday afternoon cycling session who temporarily stopped close to one of the devices to watch the race. In fact, all of these outliers were heart-rate monitors, sensors that do not go into idle mode when the bike is not moving because they are worn on the body. Figure 10 visualizes the number of unique sensors over time during the race. Since this graph contains 1 h and 45 min worth of second-level data, it condenses the peaks visually, but if zoomed in further (see Figure 11), the values appear to have a more fluid buildup when the riders move in and out of the detection range of the device. Note that, because of the large amount of occlusion inherent to the peloton, the detection zone is much smaller compared to the theoretical limit of ANT+. Also, because ANT+ devices operate mostly at 1–2 Hz (manufacturer-specific), detection failure may occur if, for example, a large and fast-moving group passes the device (only in frame for a couple of seconds), which is the case for road cycling. This graph also highlights the larger spread in lap three (Stuivenberg), where 21 participants achieved the DNF result after completing the lap. The spikes in between the peloton indicators can be attributed to smaller groups who fell behind, as well as cyclists on a recon/warm up, since after this particular race, another age group raced on the same course.

The N highest peaks were extracted from the underlying data (N corresponds to the number of laps) and labeled as the temporal trigger of the peloton passage. Ideally, a presence interval calculated around the distances before and after the camera between which sensor signals can be received should be used. Collecting accurate ground-truth data is very difficult as it depends on group size, transmit power, battery level, ambient weather information, and more. Therefore, it is more convenient to utilize the intervals extracted from the video data shown in Table 1. Since in both cases the riders are moving towards the camera, the intervals should be shifted forward because the extracted video timings do not take into account that sensor signals are still picked up when the rider is out of frame. The mean offset between the detected peak and the middle of the peloton interval was 3.8±0.9 s for the Stuivenberg section and 1.9±2.0 s for the Caudenberg section.

Due to the optimization algorithm that processes the video block before it is uploaded, a certain percentage of the video data may be discarded without information loss. A combination of varying input parameters for the algorithm is shown in Table 6. The buffer window denotes the number of seconds (video data) that need to be available before and after the timestamp of every sensor value. The quantile parameter is used to filter out sensor outliers as described above. In addition to the quantile filtering, there is also the option to filter based on the participants who registered for the study before the race. As expected, the amount of video data that could be discarded decreased when the buffer window increased. However, the rate at which it decreased was not proportional to changes to the buffer window. This was caused by the fact that some of the participants rode consistently close to each other such that the buffers needed to create their individual clips’ overlap when run through the optimizer. It is also apparent that utilizing a select group of individuals significantly reduced the amount of data that needed to be uploaded (see row of limited participants). For this particular race, the Stuivenberg device could discard 91.24% of the total video time of 1 h and 45 min while still guaranteeing 10 extra seconds before and after every sensor value. As such, it may be a good idea for organizers to input a list of ANT+ IDs that the camera should record, and it will discard values originating from sensors that are not registered before the race. The only option that the system loses is that, while participants can register retroactively, the event discovery will only work if the users specifically register their IDs before the race.

### Device Limitations

With the initial specification of the ANT+ protocol, it can only support 65,536 unique values for the device ID. Therefore, there is a real chance that, at very large events, collisions will occur between different sensors. The chance of collisions was reduced with the introduction of extended device IDs (specified in 5.2.3.1 of the ANT+ specification [36]). By using four extra bits from the transmission type, the protocol can support 1,048,576 unique values. The transmission type contains the individual transmission characteristics of a device, like the assigned page number. With the addition of the extended ID, manufacturers can rotate some of the characteristics, such as the page number, for new devices and thus increase the number of available IDs. Nonetheless, this increase in available IDs does not completely rule out collisions. In this case, the available clips on our streaming platform must aggregate multiple series of clips.

The transmission range of sensors can be affected by many variables and stating the exact severity of occlusion is therefore not possible. The radio signal might be attenuated and the range considerably reduced by obstacles such as guardrails and, more importantly, the bodies of other riders surrounding the sensor. Signals from other electrical devices and high air humidity may also negatively impact the transmission range. Therefore, the position of the antenna is vital with regard to the number of missed passages. This can be handled by placing the device higher in the air or by decoupling the antenna via a longer cable. The antenna can then be placed at a better vantage point while the camera can film from a normal point of view. Some detection failures can also be prevented by combining data from multiple devices. If a rider passes three devices in the same group but is not detected at one of the first two devices, we can use that information to assume that they also should have been in the same group at the missed passage. Another point to consider is that cyclists often have multiple sensors attached. This redundancy allows us to combine multiple clip series and compensate for missing clips if one of the sensors is not detected during a passage.

There is also a limitation imposed by the antenna regarding the number of sensor nodes it can support. The ANT USB-M module used in this research allows up to 300 nodes at a 1 Hz transmission rate in the same radio frequency space (https://www.thisisant.com/developer/components/antusb-m, accessed on 9 January 2024). This needs to be taken into consideration if the device is to be deployed in events where a very large number of participants are expected to pass the detection zone at the same time. In addition to the number of sensor-equipped participants, it should also be noted that multiple sensors per person are not uncommon in some sports.

With the range-extending part on the hLine antenna, we can control the distance at which we want to detect a sensor (±75 m vs. 4 m without obstructions). If an event required the short-ranged version (e.g., the athletics example mentioned in Section 5), a detection problem could arise if the sensors move too fast through the detection zone. Since most common sensors operate at 1–2 Hz, it could occur that the device transmits its message right before entering the detection zone and in the 0.5–1 s interval passes through the zone such that the next message is transmitted when the sensor has exited the designated zone. In this situation, it is advised to use multiple decoupled antennas spaced with a few meters in between (the exact value would be sport-specific). The device would then collect the values from all antennas and merge them together into a single source in order not to disrupt the next steps.

## 5. Conclusions and Future Work

In this paper, we presented a sensor-driven tripod that unifies sensor and video data to generate personalized highlights for participants of various sporting events wearing ANT+-capable sensors. It combines simultaneous recording of video footage and sensor data through a purpose-built device using commercially available hardware costing around EUR 115. The presence of identifiable sensor signals indicates the proximity of an individual to the device and is then used to calculate clip boundaries in the raw video stream once available on the processing server. The setup was tested with two devices along the course of a youth road-cycling race. The system achieved a detection rate of 92.9% for the study participants, generating a video clip for each instance, and was also able to accurately detect when the peloton went past the camera for each lap through the sensor signal distribution. The produced insights and content can be made available on an online platform during or after the race (based on the device configuration and upload scheme) to provide spectators with information for different POIs along the course and participants with social-media-appropriate content to share on their own platforms.

Later iterations of this work can focus more on the service delivery for users, leveraging computer vision techniques to improve the visual quality of the highlights or to generate metadata about the highlights’ content. The quality consistency of the recorded videos is currently only handled by the onboard capabilities of the camera (high dynamic range, auto focus). However, there exist techniques that can be used to improve the results in postprocessing as well. Illumination regression [37,38] or histogram equalization [39] could be appended to our pipeline and leave users more satisfied with the results. An example of the additional functions we could provide by using computer vision would be finding common athletes in a series of clips. The data would allow the system to couple a soft identity in the given context of the race. This may help to identify participants who are unable to discover their own ANT+ identifier by showing a possible visual match for the IDs (further filtered using the team jersey, etc.). We also undertook some preliminary experiments regarding smart cropping the footage. This would be useful in cases where we do not want to show the full video but only a crop of a specific region that contains the subject [33]. The challenge would then be to keep the subject in frame through a content-aware AI system. An alternative deployment option for the presented hardware could also be for fuzzy localization on smaller tracks. Some antennas, such as the hLine, can remove the range-extending part of the antenna, leaving a receiving radius of ±3 m. Covering the width of a running track in this configuration would be possible by using two devices and placing them perpendicularly on the shortest line that crosses the track. When at least one of the devices receives a signal, it would act as a kind of fuzzy gate-crossing for applications where fault tolerances of 1–2 s are allowed. For gates that cannot be covered by two devices, a more sensitive and directional antenna may be required.

## Figures and Tables

**Table 1:** Peloton passages extracted from the raw video stream. The timer lasted from when the first rider crossed the frame’s vertical center line until the last participant of the peloton crossed the same line.

	Stuivenberg		Caudenberg	
	**Start Time**	**Duration (s)**	**Start Time**	**Duration (s)**
Lap 1	13:07:18	8	-	-
Lap 2	13:17:26	6	13:13:59	11
Lap 3	13:27:35	5	13:24:13	13
Lap 4	13:38:04	7	13:34:20	7
Lap 5	13:48:05	5	13:44:26	12
Lap 6	13:58:57	5	13:55:20	6
Lap 7	14:09:56	7	14:06:06	5
Lap 8	14:20:31	5	14:16:53	6
Lap 9	14:31:38	5	14:27:51	5
Lap 10	14:41:55	6	14:38:13	7

**Table 2:** Overview of clips generated from the Stuivenberg camera. Clip duration is given in seconds. The “Abandoned” column indicates how many laps were completed. If the amount of laps equals 10, the race was completed. Absences in the table are either due to failed detection when the lap number was smaller than or equal to the abandonment value or because the participant was taken out of the race.

Device ID	Lap 1	Lap 2	Lap 3	Lap 4	Lap 5	Lap 6	Lap 7	Lap 8	Lap 9	Lap 10	Abandoned
5948	2	5	2	3	5	2	2	8	-	8	10
10435	3	5	12	4	4	8	3	3	15	5	10
19096	3	7	3	5	3	6	3	12	3	6	10
19791	5	2	2	4	6	2	5	-	5	17	10
20414	6	3	2	-	4	3	3	4	3	4	10
24554	2	4	2	2	2	5	8	2	2	4	10
33243	6	2	3	6	2	5	11	4	4	4	10
42627	13	4	11	7	5	12	7	6	14	7	10
48354	4	3	4	5	9	3	6	7	4	7	10
58537	5	-	-	2	4	2	2	2	3	-	10
22312	2	2	-	5	3	-	-	-	-	-	5
24663	-	2	2	8	16	-	-	-	-	-	5
56060	2	2	3	4	-	-	-	-	-	-	4
15454	5	9	5	-	-	-	-	-	-	-	3
42276	-	10	17	-	-	-	-	-	-	-	3
45538	21	-	-	-	-	-	-	-	-	-	1

**Table 3:** Overview of clips generated from the Caudenberg camera. Clip duration is given in seconds. The “Abandoned” column indicates how many laps were completed. If the amount of laps equals 10, the race was completed. Absences in the table are either due to failed detection when the lap number was smaller than or equal to the abandonment value or because the participant was taken out of the race.

Device ID	Lap 2	Lap 3	Lap 4	Lap 5	Lap 6	Lap 7	Lap 8	Lap 9	Lap 10	Abandoned
5948	4	2	2	5	6	13	18	9	5	10
10435	4	4	9	4	5	3	5	20	4	10
19096	3	4	-	4	4	5	2	4	3	10
19791	4	4	6	3	4	3	3	13	5	10
20414	18	12	4	6	4	5	5	3	5	10
24554	3	-	2	4	2	5	4	4	2	10
33243	7	8	2	4	6	3	4	6	2	10
42627	5	4	14	16	11	5	-	5	5	10
48354	4	5	5	3	3	3	13	3	10	10
58537	2	3	2	-	5	2	-	2	-	10
22312	2	4	3	4	-	-	-	-	-	5
24663	-	3	6	-	-	-	-	-	-	5
56060	-	5	2	-	-	-	-	-	-	4
15454	2	4	-	-	-	-	-	-	-	3
42276	25	16	-	-	-	-	-	-	-	3

**Table 4:** Clip duration outliers (Stuivenberg).

Lap	Device ID	TTP (s) *	ABD ^†^
1	42627	12.0	False
1	45538	65.0	True
3	10435	9.5	False
3	42276	263.5	True
3	42627	5.5	False
5	24663	148.5	True
6	42627	6.5	False
7	33243	6.5	False
8	19096	7.5	False
9	10435	13.5	False
9	42627	11.5	False
10	19791	71.0	Finish

* Time to peloton: number of seconds between the start of the clip and the middle of the peloton passage at the camera for that lap. ^†^ Abandonment: indicates whether the rider was taken out of the race after the lap with an outlier clip duration.

**Table 5:** Clip duration outliers (Caudenberg).

Lap	Device ID	TTP (s) *	ABD ^†^
2	20414	13.5	False
2	42276	56.5	False
3	20414	9.5	False
3	42276	201.5	True
4	42627	9.5	False
5	24663	111.0	True
5	42627	14.0	False
6	42627	8.0	False
7	5948	12.5	False
8	42627	26.0	False
8	48354	12.0	False
8	5948	18.0	False
9	10435	15.5	False
9	19791	9.5	False
10	48354	6.5	Finish

* Time to peloton: number of seconds between the start of the clip and the middle of the peloton passage at the camera for that lap. ^†^ Abandonment: indicates whether the rider was taken out of the race after the lap with an outlier clip duration.

**Table 6:** Overview of optimization schemes with differing initialization parameters. Values denote the percentage of video footage that can be discarded without losing clip duration for the given buffer window.

	Stuivenberg			Caudenberg		
Buffer window (in seconds)	5	10	20	5	10	20
Quantile = 1.0	26.84%	17.02%	4.73%	45.03%	35.98%	24.10%
Quantile = 0.9	54.40%	41.37%	24.08%	52.33%	42.52%	29.10%
Quantile = 0.8	57.59%	44.68%	27.08%	57.30%	46.87%	32.86%
Limited to study participants (N = 16)	94.17%	91.24%	84.75%	93.05%	89.79%	84.56%

## Data Availability

Publicly available datasets were analyzed in this study. These data can be found at: https://github.com/robbedec/datasets/tree/master/STRADA/lierde, accessed on 15 November 2023.
